# Prevalence of and Trends in Diabetes Among Veterans, United States, 2005–2014

**DOI:** 10.5888/pcd14.170230

**Published:** 2017-12-14

**Authors:** Ying Liu, Sonica Sayam, Xiaonan Shao, Kesheng Wang, Shimin Zheng, Ying Li, Liang Wang

**Affiliations:** 1 Department of Biostatistics and Epidemiology, East Tennessee State University, Johnson City, Tennessee; 2 Carnegie Mellon University, Pittsburgh, Pennsylvania; 3 Department of Environmental Health, East Tennessee State University, Johnson City, Tennessee

## Abstract

Diabetes is a highly prevalent chronic disease among US adults, and its prevalence among US veterans is even higher. This study aimed to examine the prevalence of and trends in diabetes in US veterans by using data from the US National Health and Nutrition Examination Survey from 2005 through 2014. The overall prevalence of diabetes and undiagnosed diabetes was 20.5% and 3.4%, respectively, and increased from 15.5% in 2005–2006 to 20.5% in 2013–2014 (*P* = .04). Effective prevention and intervention approaches are needed to lower diabetes prevalence among US veterans and ultimately improve their health status.

## Objective

Diabetes was the seventh leading cause of death in the United States in 2013 (1). Approximately 30.3 million Americans had diabetes, including an estimated 7.2 million who had the disease but had not received a diagnosis (2). Diabetes is associated with multiple chronic conditions, including cardiovascular diseases, stroke, and disorders leading to amputation. The estimated annual cost of diabetes on the US health care system overall is $245 billion (2,3).

Diabetes is more prevalent among US veterans, who make up 9% of the civilian US population, than among the general population and affects nearly 25% of US Department of Veterans Affairs (VA) patients (4,5). The objective of this study was to assess the prevalence of diabetes among US veterans by using data from the National Health and Nutrition Examination Survey (NHANES).

## Methods

We used data from 5 NHANES cycles conducted from 2005 through 2014. NHANES uses a stratified multistage probability sampling approach to obtain representative samples from 50 states and the District of Columbia. Veteran status was self-identified through participant household interviews. The unweighted sample size for 2013–2014 was 491 and ranged from 472 to 685 for each cycle from 2005 through 2012.

Diabetes in a participant was defined as having at least 1 of 4 conditions: 1) a glycated hemoglobin A_1c_ of 6.5% or higher, 2) fasting plasma glucose of 126 mg/dL or higher, 3) a 2-hour plasma glucose of 200 mg/dL or higher, or 4) a diagnosis of diabetes by a physician or other health care provider (2). We defined people without diabetes as those who had none of these conditions. A person with a body mass index (BMI; defined as the weight in kilograms divided by the square of the height in meters) of 30 or higher was classified as obese (2).

Demographic variables were age, sex, and race/ethnicity (white, African American, Hispanic, and other race). Age was sorted into 3 groups: 20 to 44 years, 45 to 64 years, and 65 years or older. Socioeconomic status included poverty level and education level. Poverty level is a ratio of annual income to the federal poverty level (FPL) adjusted for the number of people in the household and where they lived. We categorized poverty level into 3 groups: less than 100% of FPL, 100% to less than 300% of FPL, and 300% or more of FPL. Education was categorized by years of education: less than 12 years, 12 years, and more than 12 years.

Rao-Scott χ^2^ test measured the bivariate association of diabetes and each exploratory variable. We used the Cochran-Armitage trend test to assess prevalence of diabetes time-trends from 2005 through 2014, and the proportional test was used to compare prevalence differences. Significance was set at *P* less than .05. All analyses were performed with SAS version 9.4 (SAS Institute, Inc).

## Results

The overall pooled weighted prevalence of diabetes in NHANES for 2013–2014 was 20.5% (95% confidence interval [CI], 15.9–25.2%), and the prevalence of undiagnosed diabetes was 3.4% (95% CI, 1.1%–5.6%), (Table 1). Diabetes was most prevalent among veterans aged 65 years or older (27%), among male veterans (22%), among veterans with less than 12 years of education (33.5%) and among veterans with an annual income below the 100% FPL (23.8%). The highest prevalence of obesity was among veterans aged 45 to 64 years (53.1%), male veterans (41.1%), veterans with less than 12 years of education (51.4%), and veterans living below the 100% FPL (47.2%). Poverty level (*P* = .005) and education (*P* = .03) were significantly associated with the odds of diabetes. The highest prevalence of diabetes (25.7%) and obesity (43.5%) was observed among Hispanic veterans.

**Table 1:** Weighted Prevalence of Diabetes and Obesity in US Veterans Aged 20 Years or Older, National Health and Nutrition Examination Survey, 2013–2014

Variable (n^a^)	Diabetes^b^, % (95% CI)	*P* Value^c^	Undiagnosed Diabetes^b^, % (95% CI)	*P* Value^c^	Obesity^d^, % (95% CI)	*P* Value^c^
**Overall (491)**	20.5 (15.9–25.2)	NA	3.4 (1.1–5.6)	NA	40.7 (35.0–46.4)	NA
**Age, y**						
22–44 (78)	4.8 (0.0–10.4)	<.001	NA^e^	<.001	31.0 (18.6–43.3)	<.001
45–64 (156)	20.8 (12.5–29.1)		4.5 (0.0–9.5)		53.1 (42.5–63.7)	
≥65 (257)	27.0 (19.7–34.3)		4.0 (0.8–7.3)		36.3 (28.4–44.2)	
**Sex**						
Male (459)	22.0 (17.0–27.0)	<.001	3.7 (1.2–6.1)	.01	41.1 (35.2–47.1)	.68
Female (32)	3.9 (0.0–8.8)		NA^e^		35.8 (13.4–58.3)	
**Race**						
White (294)	20.3 (14.6–25.9)	.79	3.7 (0.9–6.5)	.34	40.7 (33.8–47.7)	.07
Black (125)	22.8 (14.8–30.7)		2.1 (0.0–4.5)		40.6 (30.9–50.3)	
Hispanic (47)	25.7 (12.8–38.6)		3.1 (0.0–7.9)		43.5 (26.8–60.2)	
Other (25)	11.6 (0.0–23.5)		1.2 (0.0–3.7)		35.4 (9.1–61.6)	
**Federal poverty level**						
<100% (62)	23.8 (7.7–39.8)	.005	2.1 (0.0–4.6)	.30	47.2 (30.1–64.4)	.08
100% to <300% (216)	21.3 (14.1–28.4)		3.06 (0.0–6.5)		42.8 (33.9–51.6)	
≥300% (213)	19.4 (12.9–26.0)		3.8 (1.8–7.3)		37.9 (29.7–46.2)	
**Education, y**						
<12 (45)	33.5 (11.5–55.6)	.03	2.0 (0.0–4.8)	.25	51.4 (29.9–72.6)	.70
12 (117)	18.5 (9.9–27.1)		2.7 (0.0–5.8)		42.2 (29.5–54.9)	
>12 (329)	19.9 (14.4–25.4)		3.68 (0.7–6.7)		39.2 (32.5–45.9)	

Abbreviations: CI, confidence interval; NA, not applicable.

a Unweighted total number of each category.

b Weighted prevalence of total diabetes cases. The presence of diabetes is defined as any participant who had at least 1 of 4 conditions: 1) a glycated hemoglobin A_1c_ of 6.5% or higher, 2) fasting plasma glucose of 126 mg/dL or higher, 3) a 2-h plasma glucose of 200 mg/dL or higher, or 4) a diagnosis of diabetes by a physician or other health care provider.

c *P* values calculated by using Rao–Scott χ^2^ test for bivariate association.

d Weighted prevalence of obesity (body mass index, defined as weight in kilograms divided by height in m^2^) ≥30.

e Zero cases in the data set.

The overall prevalence trend of diabetes increased from 15.5% in 2005–2006 to 20.5% in 2013–2014 (*P* = .04 for trend test) and peaked in 2009–2010 (22.6%), (Table 2). The prevalence increased significantly among male veterans, from 16.5% in 2005–2006 to 22.0% in 2013–2014 (*P* = .04 for trend test). The prevalence of diabetes among veterans who had less than 12 years of education increased from 21.9% in 2005–2006 to 33.5% in 2013–2014 (*P* = .04 for trend test). Among veterans with more than 12 years of education, the prevalence increased from 12.3% in 2005–2006 to 19.9% in 2013–2014 (*P* = .03 for trend test). The increase in age-standardized prevalence was not significant for any of the 3 age groups.

**Table 2:** Weighted Prevalence of Diabetes^a^ in US Veterans Aged 20 Years or Older, National Health and Nutrition Examination Survey, 2005–2014

Variable	2005–2006 (n = 622)	2007–2008 (n = 670)	2009–2010 (n = 685)	2011–2012 (n = 472)	2013–2014 (n = 491)	*P* Value for Trend^b^
**No. with diabetes^c^**	121	157	183	124	111	NA
**Overall prevalence**	15.5 (12.4–18.7)	20.0 (16.3–23.7)	22.6 (19.0–26.3)	20.8 (16.0–25.7)	20.5 (15.9–25.2)	.04
**Age, y**						
22–44	5.2 (0.4–9.7)	4.6 (0.0–10.3)	4.2 (0.3–8.2)	9.3 (0.0–19.3)	4.8 (0.0–10.4)	.50
45–64	10.7 (6.2–15.1)	17.8 (11.8–23.8)	21.1 (14.5–27.7)	14.1 (7.2–20.9)	20.8 (12.5–29.1)	.06
≥65	26.5 (20.7–32.3)	30.9 (24.9–36.9)	34.3 (28.5–40.1)	34.0 (25.8–42.2)	27.0 (19.7–34.3)	.10
**Sex**						
Male	16.5 (13.2–19.9)	21.5 (17.5–25.4)	24.1 (20.2–27.9)	22.1 (16.9–27.3)	22.0 (17.0–27.0)	.04
Female	1.6 (0.0–5.0)	6.3 (0.0–14.7)	6.3 (0.0–12.9)	7.0 (0.0–16.0)	3.9 (0.0–8.8)	.14
**Race/ethnicity**						
White	15.0 (11.4–18.6)	20.9 (16.5–25.2)	22.8 (18.5–27.2)	19.0 (13.4–24.5)	20.2 (14.6–25.9)	.16
Black	20.4 (13.4–27.5)	18.8 (12.1–25.5)	19.8 (13.3–26.2)	26.4 (18.6–34.1)	22.8 (14.8–30.7)	.25
Hispanic	25.3 (7.3–43.2)	12.34 (5.1–19.6)	28.26 (5.3–38.7)	15.5 (5.7–25.2)	25.7 (12.8–38.6)	.16
Other	2.0 (0.0–6.6)	10.5 (0.0–28.0)	19.1 (0.0–39.2)	39.3 (6.5–72.2)	11.6 (0.0–23.5)	.11
**Poverty level**						
<100%	14.7 (5.3–24.1)	23.7 (12.3–35.1)	12.6 (3.4–31.8)	21.2 (9.8–32.6)	23.8 (7.7–39.8)	.27
100% to <300%	21.2 (15.6–36.8)	19.1 (14.1–24.1)	29.0 (22.6–35.4)	27.0 (18.2–35.7)	21.3 (14.1–28.4)	.22
≥300%	11.7 (7.6–15.7)	20.3 (14.6–26.0)	19.9 (15.3–24.6)	16.65 (10.3–23.0)	19.4 (12.9–26.0)	.06
**Education, y**						
<12	21.9 (12.9–30.8)	18.1 (11.1–25.0)	23.2 (13.9–32.5)	32.1 (17.4–46.9)	33.5 (11.5–55.6)	.04
12	19.3 (12.5–26.1)	28.1 (19.9–36.3	25.6 (17.8–33.5	16.0 (6.9–25.1)	18.5 (9.9–27.1)	.43
>12	12.3 (8.5–16.1)	17.1 (12.3–21.8)	21.31 (16.7–25.9)	20.51 (14.4–26.7)	19.9 (14.4–25.4)	.03

Abbreviation: NA, not applicable.

^a^ The presence of diabetes is defined as any participant who had at least 1 of 4 conditions: 1) a glycated hemoglobin A_1c_ of 6.5% or higher, 2) fasting plasma glucose of 126 mg/dL or higher, 3) a 2-h plasma glucose of 200 mg/dL or higher, or 4) a diagnosis of diabetes by a physician or other health care provider.

^b^ *P* values calculated by using Cochran–Armitage trend test.

^c^ Unweighted number of cases of diabetes.

## Discussion

Diabetes is more prevalent among US veterans than among the general population (3,5). This high prevalence is primarily attributable to the high prevalence of obesity among this population (5). Obesity and diabetes are genetically linked (6,7). People with obesity are more prone to the major contributors to type 2 diabetes — insulin resistance and β cell dysfunctions (8,9).

Diabetes is preventable for many people and treatable, but people with undiagnosed diabetes have delays in receiving effective treatment and, thus, are more likely to develop complications such as coronary artery diseases and retinal microvascular diseases than people with diabetes who receive treatment (10). However, in the United States the proportion of people with diabetes who had diabetes diagnosed decreased from 72.5% in 2005–2008 to 67.7% in 2009–2012 (7).

Overall prevalence of diabetes in the NHANES participants increased from 15.5% in 2005–2006 to 20.0% in 2007–2008 and remained stable at around 20.0% in NHANES surveys from 2009 through 2014. This change is partially because fewer people aged 65 years or older were included in the 2005–2006 survey (37.8%) than that in the 2007–2014 surveys (>40%). Unlike previously reported findings (3), our findings showed that the prevalence of diabetes by poverty level did not decline with increasing income, and this trend persisted over time. This persistence may be due to a small number diabetes cases and some unidentified confounders. The underlying reasons for this trend merit further investigation.

Our study had limitations. Statistical tests cannot be applied to data for female veterans because NHANES reported few or no female veterans with the disorder. In addition, NHANES data do not distinguish between type 1 and type 2 diabetes or gestational diabetes in the NHANES participants.

Certain factors limit the accuracy of the estimated prevalence of diabetes in US veterans when using VA databases. First, based on the eligibility criteria and capability of VA facilities, in fiscal year 2014 less than 30% of the total veteran population sought VA health care, and more than 70% of veterans sought care outside the VA system even though some of them were enrolled in the VA system (10). NHANES uses a complex, multistage, probability sampling design, which allows selected participants to represent noninstitutionalized US civilians. Consequently, our study sample represents US noninstitutionalized veterans. Therefore, NHANES is an appropriate resource to provide complementary results for VA data, and our findings can be used to determine the prevalence of diseases in the veteran population. Future research should combine nationwide data with VA data to obtain estimates that are more accurate. In spite of these limitations, the high prevalence of diabetes reported here calls for cost-effective strategies for prevention and intervention for US veterans.
